# Cysteine proteases as digestive enzymes in parasitic helminths

**DOI:** 10.1371/journal.pntd.0005840

**Published:** 2018-08-23

**Authors:** Conor R. Caffrey, Louise Goupil, Karina M. Rebello, John P. Dalton, David Smith

**Affiliations:** 1 Center for Discovery and Innovation in Parasitic Diseases, Skaggs School of Pharmacy and Pharmaceutical Sciences, University of California San Diego, La Jolla, California, United States of America; 2 Department of Biology, University of San Francisco, San Francisco, California, United States of America; 3 Laboratório de Toxinologia and Laboratório de Estudos Integrados em Protozoologia, Instituto Oswaldo Cruz (Fiocruz), Rio de Janeiro, Brazil; 4 School of Biological Sciences, Medical Biology Centre, Queen´s University Belfast, Belfast, United Kingdom; Walter and Eliza Hall Institute of Medical Research, AUSTRALIA

## Abstract

We briefly review cysteine proteases (orthologs of mammalian cathepsins B, L, F, and C) that are expressed in flatworm and nematode parasites. Emphasis is placed on enzyme activities that have been functionally characterized, are associated with the parasite gut, and putatively contribute to degrading host proteins to absorbable nutrients [1–4]. Often, gut proteases are expressed as multigene families, as is the case with *Fasciola* [5] and *Haemonchus* [6], presumably expanding the range of substrates that can be degraded, not least during parasite migration through host tissues [5]. The application of the free-living planarian and *Caenorhabditis elegans* as investigative models for parasite cysteine proteases is discussed. Finally, because of their central nutritive contribution, targeting the component gut proteases with small-molecule chemical inhibitors and understanding their utility as vaccine candidates are active areas of research [7].

## Angiostrongylus

Cysteine proteases are essential in the degradation of host hemoglobin, the principal source of amino acids for many parasitic helminths. *Angiostrongylus cantonensis* and *A*. *costaricensis* are the etiological agents of abdominal angiostrongyliasis and eosinophilic meningoencephalitis, respectively [8, 9]. This nematode cycles between rodents and mollusks, and the parasite infects humans via the ingestion of raw vegetables and/or mollusks containing third-stage larvae (L3). Only six cysteine protease sequences are available in the GenBank protein database for adult *Angiostrongylus*. The function(s) of these putative proteins is still unknown; however, they may play a role in immune system evasion and nutrient acquisition. In *A*. *cantonensis*, cathepsin B cysteine proteases may contribute to the invasion of the central nervous system during parasite–host interactions [10], penetration into the host’s gut wall [11], and feeding [12, 13]. Reverse-transcription PCR (RT-PCR) revealed that cysteine protease transcripts of *A*. *cantonensis* are present in both larval and adult worms [14]. In addition, a proteolytic enzyme was localized in the intestine of juvenile and adult worms, indicating its probable contribution to feeding and nutrition [14, 15].

## Hookworm and *Haemonchus*

The two most prevalent hookworm species infecting humans are *Necator americanus* and *Ancylostoma duodenale*, with *A*. *ceylanicum* being found in certain areas [16, 17]. *Haemonchus contortus*, the barber’s pole worm, which is common in pastures where cattle, sheep, and goats are farmed, is an appropriate comparator for human hookworms, being also hematophagous with a similar life history. Third-stage larvae (L3) are found in contaminated feces and are either ingested (*Haemonchus* and *A*. *duodenale*) or can actively invade the skin (hookworms). Adult parasites settle, mature, and mate in the abomasum (*Haemonchus*) or duodenum (hookworms). Adults are hematophagous, with blood being a rich source of nutrients, not least for the prodigious production of eggs: female parasites can produce 10,000–28,000 eggs per day ([18, 19] and references therein).

Considerable transcriptional effort is made by these nematodes into expressing a series of cathepsin B-like proteases that are found in the parasite’s gut (esophagus and cecum) [6, 20–22]. These enzymes, among other protease classes [23], degrade blood proteins to absorbable nutrients [3, 4, 24–27] and are targets for small-molecule drug discovery (see below). Based on both classical biochemical and recent transcriptomic/genomic evidence, *N*. *americanus* and *A*. *ceylanicum* express six gut cysteine proteases [4, 22, 28], and *Haemonchus* as many as 22 individual proteases ([29] and references therein). In the latter case, further complexity is introduced by geographical variation in the molecular sequences and the activity of the individual proteases [30–33]. Differences in proteases are also apparent for the same parasite strains that are isolated from different hosts [33, 34]. Because they operate at the interface between host and parasite, the cysteine proteases of gastrointestinal nematodes have been tested as vaccine candidates with varying degrees of efficacy [24, 33, 35, 36]. In the case of *Haemonchus*, defining exactly which proteases represent vaccine candidates is complicated by the geographic and strain variations alluded to above.

## *Trichuris* and *Ascaris*

*Trichuris* (whipworm) and *Ascaris* (giant roundworm), together with hookworm, comprise the three most prevalent soil-transmitted helminths, which collectively infect approximately 1.5 billion people [37]. *T*. *muris* (murine whipworm) and *T*. *suis* (porcine whipworm) are employed as models of *T*. *trichiura* (human whipworm). *A*. *lumbricoides* and *A*. *suum* are widespread roundworms of humans and pigs, respectively, with *A*. *suum* serving as a model [37, 38].

Drake and colleagues (1994) [39] first described a cysteine protease activity in soluble extracts of *T*. *muris* adult worms using a fluorogenic peptidyl substrate. They suggested a contribution by this enzyme in nutrition and/or invasion. A cysteine protease similar to cathepsin B was subsequently characterized in adult *T*. *suis* gut extracts and excretory/secretory products (ESP) using both fluorogenic peptide substrates and protein substrate gels [40]. Again, this enzyme was postulated to be involved in nutrition.

Hasnain and colleagues [41] showed experimentally that proteolytic activity in *T*. *muris* ESP is responsible for degrading host intestinal mucin. Whereas serine protease activity appeared to be the most active, the authors also suggested that cysteine proteases present in ESP contribute to disrupting the polymeric mucin network. High-throughput transcriptomics data indicate that a suite of cysteine proteases are expressed in *T*. *muris*, including legumain; calpains; and cathepsins B, L, F, and Z. [42]. One or more of these may be involved in digestion. Recently, Santos and colleagues reported transcripts encoding gut-specific cysteine proteases in extracts of adult *T*. *trichiura* [43], and, at the proteome level, a number of these possessed immunomodulatory activity [44].

Few studies have focused on the functional characterization of *Ascaris* cysteine proteases. Early reports described a hemoglobinolytic cysteine protease activity in *A*. *suum* lysates [45, 46]. In addition, protease activity was described in *A*. *suum* intestinal lysates using a fluorescently (bodipy)-labeled casein substrate, although no significant inhibition with the cysteine protease inhibitors, E-64 and iodoacetamide, was measured [47]. In contrast, aspartic proteases seem to represent the largest group of *A*. *suum* proteases involved in digestion [47], whereas cysteine proteases are a relatively minor constituent. Finally, the predicted secretome of *A*. *suum* includes five cysteine protease sequences that may contribute to feeding and/or degradation of host tissue [48].

## Schistosoma

Schistosomiasis, or bilharzia, is caused by several parasitic flatworms of the genus *Schistosoma*. It is endemic in over 70 tropical/subtropical countries and infects more than 200 million people. Infective larvae (cercariae) are released from intermediate aquatic snail hosts, penetrate the outer and inner dermal skin layers, and then, as schistosomula, migrate to the blood capillaries. Schistosomula are carried to the lungs and subsequently to the liver before settling as mated male and female worms in the mesenteric (*S*. *mansoni* and *S*. *japonicum*) or bladder (*S*. *haematobium*) venules. Blood proteins, particularly hemoglobin, are an essential source of nutrients with which the female worms produce hundreds of eggs per day. A portion of the eggs laid exit the host via the intestine or bladder to continue the life cycle; however, many become trapped in the liver, gastrointestinal tract, or urinary tissues, where they can induce the immunopathology and fibrosis that are often associated with schistosomiasis [49].

Timms and Bueding (1959) [50] first described the presence of proteases with an acidic pH optimum in schistosome extracts. They suggested a role for these in degrading hemoglobin to free amino acids in the schistosome gut and also highlighted their potential as drug targets. Today, we know that an array of proteases are secreted into the schistosome gut, including papain-like cysteine proteases (clan CA, family C1) cathepsins B1, L1/F, L2, L3, and C (Table 1), as well as an aspartic protease, cathepsin D (Clan AA) [51–53].

**Table 1:** Helminth parasite cysteine proteases and their functions.

Parasite	Protease	Proposed Function	References
**Nematodes**			
***Angiostrongylus cantonensis***	Cathepsin B	Tissue invasion	[10]
	Cathepsin B1	Degradation of hemoglobin and IgG	[14]
***Ancylostoma caninum***	Cathepsin B1	Degradation of tissues	[26, 151]
	Cathepsin B2	Degradation of hemoglobin	[26, 151, 152]
***Haemonchus contortus***	Cathepsin B (multiple)	Degradation of hemoglobin, fibrinogen, collagen, and IgG	[151]
	Cathepsin L	Degradation of hemoglobin, fibrinogen, collagen, and IgG; blood anticoagulation	[34]
***Necator americanus***	Cathepsins B2, B3, B4 and B5	Degradation of globin fragments and gelatin	[4]
***Trichuris muris***	Gut cysteine protease	Digestion	[42, 43]
	Cathepsin B-like	Digestion	[40]
***Ascaris suum***	Cathepsin B-like	Digestion	[47]
**Trematodes**			
***Schistosoma haematobium***	Cathepsin B	Degradation of hemoglobin	[153]
***Schistosoma japonicum***	Cathepsin B	Tissue invasion	[154]
	Cathepsin C	Degradation of hemoglobin	[155]
	Cathepsin L1	Degradation of hemoglobin; membrane turnover	[64, 156]
***Schistosoma mansoni***	Cathepsin B1	Degradation of hemoglobin, albumin, and IgG	[56]
	Cathepsin B2	Tegumental protein turnover; degradation of endocytosed proteins; parasite protection	[157]
	Cathepsin C	Maturation of other parasite cysteine proteases	[56]
	Cathepsin L1	Degradation of hemoglobin	[63]
	Cathepsin L2	Location in ovovitelloduct and uterus suggests functions associated with egg production, e.g., alteration of fluid viscosity to aid egg transport and/or activation of phenoloxidase, which crosslinks eggshell proteins; degradation of hemoglobin	[65, 66]
	Cathepsin L3	Degradation of hemoglobin and albumin	[52]
***Trichobilharzia regenti***	Cathepsin B	Degradation of myelin protein	[81]
***Clonorchis sinensis***	Cathepsins B1, B2, B3 and B4	Degradation of gelatin, hemoglobin, albumin, fibronectin, and IgG	[82]
	Cathepsins F4, F6 and F11	Degradation of hemoglobin, albumin, collagen, and IgG	[86]
***Paragonimus westermani***	Cathepsin L-like	Degradation of extracellular matrix proteins and IgG	[98, 158]
	Cathepsin F	Possible role in egg production	[159]
	Hemoglobinase	Degradation of hemoglobin	[100]
***Fasciola hepatica***	Cathepsins B1 and B3	Penetration of the intestinal wall	[104, 109]
	Cathepsin B2	Penetration of the intestinal wall; degradation of albumin and IgG	[104, 109, 160]
	Cathepsin C	Degradation of peptides	[101]
	Cathepsin L1	Degradation of hemoglobin, IgG, and TLR3	[101, 102, 107, 108]
	Cathepsin L2	Degradation of hemoglobin, collagen degradation, and IgG	[101, 107]
	Cathepsin L3	Degradation of collagen	[102]
	Cathepsin L5	Degradation of hemoglobin, fibronectin, laminin, and IgG	[101, 105]
**Cestoda**			
***Taenia solium***	Cathepsin L	Degradation of tissues and IgG	[110, 111]
***Taenia pisiformis***	Cathepsin L	Degradation of IgG and fibronectin	[112]
***Echinococcus multilocularis***	Cathepsin L and B	Degradation of IgG, albumin, collagen, and fibronectin	[118, 119]

**Abbreviations**: IgG, immunoglobulin G; TLR3, toll-like receptor 3.

The *S*. *mansoni* cathepsin B1 (SmCB1, also known as Sm31) was first described and characterized as a potential diagnostic protein based on potent immunogenicity in mice and humans [54–61]. The first described *S*. *mansoni* cathepsin L, SmCL1 [62–64], was later revealed to be more related to cathepsins F, which are distinguished from other cathepsins by various structural motifs and an N-terminal propeptide extension [1]. SmCL2, in contrast, is a defined cathepsin L, and because it was detected in the reproductive system of female schistosomes, it was speculated to function in increasing the fluidity of proteins in secretions within the ovovitelloduct and activation of phenoloxidase, an enzyme involved in crosslinking eggshell proteins [65]. Bogitsh and colleagues [66] showed, using monospecific polyclonal antibodies, that both SmCL1/F and SmCL2 are located in the schistosome digestive tract and are likely digestive enzymes. More recently, Dvořák and colleagues [52] discovered a third and distinct *S*. *mansoni* cathepsin L, SmCL3, which is also localized in the gastrodermal cells.

Digestion of host blood proteins by schistosomes is performed in a bifurcated gut, which does not possess an anus, and hence the proteases and degraded contents are emptied by regular regurgitation into the blood stream of the host. The pH within the gut is in the range of 4.0 to 6.5 [67], which is important in regulating the digestive function of the gut proteases. Delcroix and colleagues [67] described how a “network” of proteases function best in acid pH and postulated that they either act simultaneously or in some specifically ordered manner to reduce protein macromolecules to absorbable peptides and amino acids. The pH of the gut is also important in facilitating denaturation of the protein substrates, unfolding their tertiary structure to make them more prone to hydrolysis [1, 67].

Immunolocalization studies suggest that the cysteine proteases are synthesized by the gastrodermal cells and are secreted within vesicles into the gut lumen as inactive precursors or “zymogens.” Therefore, another important contribution of the gut’s low pH is the activation of these zymogens to their mature catalytic forms. Recombinant zymogen forms of the SmCL1/F, SmCL2, and SmCL3 effectively autocatalytically activate in vitro to mature enzymes only in low pH solutions [53]. SmCB1 is different in this respect, as the zymogen does not autocatalytically activate at low pH in vitro [53, 56].

An alternative mechanism for activating the SmCB1 zymogen was discovered, whereby a specific cleavage site at the junction between the propeptide and mature enzyme domain is processed by an asparaginyl endopeptidase (SmAE or legumain), a cysteine protease that is also found in the parasite gut [56, 68]. Sajid and colleagues [56] showed experimentally that SmAE could activate SmCB1 in vitro. However, paradoxically, when RNA interference (RNAi) methods were used to knock out SmAE activity in live schistosomula, SmCB1 was still fully processed and activated, suggesting that SmAE is not essential to activate SmCB1 in vivo [69]. Subsequently, X-ray crystal structures of the zymogen, mature enzyme, and an inactive intermediate form of SmCB1, resolved by Jílková and colleagues [70], highlighted a further mechanism for protease activation. They showed that the propeptide of the SmCB1 contains a unique alpha-helix, alpha-3p. This helix is positioned upstream of the propeptide–mature enzyme juncture, protrudes from the enzyme core, and interacts with sulfated polysaccharides like dextran sulfate and heparin sulfate. Binding of sulfated polysaccharides to the alpha-3p was found to be essential for complete processing of the SmCB1.

The action of the cysteine and aspartic protease network in the schistosome gut would produce a plethora of peptides of various lengths that must be further broken down into free amino acids before they can be utilized in protein anabolism by the parasite. Dipeptidyl peptidase (DPP) I and III activities have been described in adult *S*. *mansoni* [71, 72]. Also, *S*. *japonicum* DPP I, a cathepsin C cysteine protease, was functionally expressed and shown to process dipeptides [71]. It is assumed that this enzyme plays a role in the hydrolysis of peptides alongside a leucine aminopeptidase (SmLAP) [73] to release free amino acids. Another possible role for DPP I is in assisting SmAE in the activation of proteases by trimming off propeptide amino acids that remain following processing by SmAE [1].

The redundancy of protease activation mechanisms (autocatalytic, *trans* activation/trimming, and sulfate polysaccharide triggered) within the schistosome gut probably reflects the central importance of the protease network in the nutrition, survival and reproduction of the parasite. Previous studies have demonstrated the validity of these enzymes as drug targets [74–76], with Abdulla and colleagues [74] demonstrating that treatment with the vinyl sulfone cysteine protease inhibitor, K11777, reduces worm and egg burden in mice. Furthermore, Ricciardi and colleagues [77] have shown that vaccination with recombinant SmCB1 induces strong protection against infection in a murine model of *S*. *mansoni* infection. The more we learn about the physicochemical, structural, and biological properties of these critical proteases, the better we will be able to design novel antischistosome drugs or vaccines.

## Trichobilharzia

The schistosomes *Trichobilharzia regenti* and *T*. *szidati* can only complete their life cycles in specific birds. However, their cercariae can penetrate nonspecific host (mammalian) skin (giving rise to cercarial dermatitis in humans) and migrate for a time through various tissues before dying [78]. An ortholog of SmCB1 has been characterized in the gut of both *T*. *regenti* and *T*. *szidati* schistosomula [79, 80] with at least 6 isoforms of TrCB1 (1.1–1.6) identified by PCR in the former species [81]. Interestingly, when recombinantly expressed, isoforms 1.1 and 1.4 efficiently degraded myelin basic protein but not hemoglobin, consistent with *T*. *regenti*’s tropism for and migration along the nervous system [81]. Like the purified zymogen of SmCB1 (above), the TrCB1.1 zymogen requires processing in *trans* (e.g., by SmAE) to become fully active, whereas the TcCB1.4 zymogen can autoprocess its own maturation. Like SmCB1 [60], TrCB1 is released from the parasite and may be useful as a serodiagnostic marker of infection [80].

## Clonorchis

*Clonorchis sinensis*, the oriental liver fluke, is a food-borne parasite that inhabits the intrahepatic bile duct and causes clonorchiasis [82]. Humans are infected by eating raw or undercooked freshwater fish contaminated with *C*. *sinensis* metacercariae. Upon ingestion, the excysted metacercariae and the juvenile flukes invade the duodenum and migrate to the intrahepatic bile ducts, where they develop into adults [83].

Cysteine proteases (cathepsins B, F, and L and legumain) are expressed in different stages of the *C*. *sinensis* life cycle, with up-regulation occurring in adults [84–86]. Transcriptomic and proteomic analysis of *C*. *sinensis* tissues and ESP has shown that these cysteine proteases make up a major portion of the parasite’s total protease complement (especially cathepsin F proteases (CsCFs) [86–89]. Furthermore, the cysteine proteases represent the most highly expressed protease family across multiple *C*. *sinensis* life stages associated with the mammalian host. This is indicative of *C*. *sinensis’* dependency on these proteases during infection. Immunolocalization studies in adult parasites have shown that the cathepsins F and L proteases are mainly associated with the parasite’s intestinal wall and lumen [86, 90–92]. These findings and the fact that *C*. *sinensis* cysteine proteases can degrade an array of host proteins (e.g., hemoglobin, albumin, IgG, collagen, gelatin, fibronectin, and laminin) suggest functions in nutrition in addition to immune system evasion and tissue invasion [82, 90, 93]. The tandem expression and colocalization of cysteine protease inhibitors (cystatins) in this parasite suggests that the cysteine proteases are finely regulated [85, 94, 95]. The balance between cysteine proteases and cystatins [94, 95] could be susceptible to disruption by cysteine protease-inhibiting drugs.

## Paragonimus

The lung fluke *Paragonimus westermani* causes paragonimiasis. Humans become infected with *P*. *westermani* after consuming raw or inadequately cooked crustaceans and/or wild boar meat that contain metacercariae. These metacercariae invade the duodenum and migrate through the abdominal wall before reaching the lungs and maturing as adult worms [96].

Cysteine proteases in *P*. *westermani* facilitate tissue invasion and immune evasion as well as nutrient acquisition [97, 98]. These enzymes are expressed in different developmental stages of *P*. *westermani*, especially in the secretome of adult flukes, consistent with their contribution to digestion of host proteins. Moreover, with at least 15 cysteine proteases present in the *P*. *westermani* secretome as well as their relatively high overall expression, cysteine proteases represent the major proteins secreted by this parasite [97, 99]. Despite a dearth of biochemical characterizations for *P*. *westermani* cysteine proteases, Choi and colleagues [100] have identified and characterized a hemoglobinase cysteine protease in adult worms. The application of a cysteine protease inhibitor, E-64, not only inhibits parasite cysteine proteases in vitro but also prevents *P*. *westermani* peritoneal invasion [98]. This suggests that cysteine protease inhibitors may prove valuable as a novel chemotherapeutic approach for the treatment of paragonimiasis.

## Fasciola

Recent genomic, transcriptomic, and proteomic analyses of the *Fasciola hepatica* life stages that infect the mammalian host have provided valuable information on the expression of key proteases potentially involved in host–parasite interactions [5, 101]. These data have also enabled clarification of the cysteine protease family structure, i.e., the cathepsins L represent a monophyletic family that contains five distinct clades, each with varying numbers of members: FhCL1 (6), FhCL2 (1), FhCL3 (5), FhCL4 (2), and FhCL5 (3). In contrast, the cathepsin B family consists of a monophyletic group with a single clade of 7 members [5].

These virulence-associated cysteine proteases show a strict temporal regulation in their expression during the parasite’s development in the mammalian host, which has furthered our understanding of their function [5]. For example, FhCL3 is highly expressed in newly excysted juveniles (NEJ) that initiate infection, but once the parasite has traveled across the intestinal wall, its expression is then down-regulated [5, 101]. Following this, the expression of FhCL1, FhCL2, and FhCL5 is up-regulated as the parasite migrates through the liver parenchyma and then takes up residence within the bile ducts [5, 101]. FhCL3 can accommodate small Gly residues in its active-site S3 pocket and bulky Pro residues in its S2 pocket, thereby having a potent ability to degrade collagen (which contains repeat Gly-Pro-X motifs). This feature allows the parasite to rapidly traverse the intestinal tissue and penetrate the Glisson’s capsule of the liver [102, 103]. NEJs at this stage are considered tissue feeders rather than blood feeders, so, besides the primary role of FhCL3 in tissue invasion, this enzyme may also be important for feeding [103]. Although FhCL2 has less collagenolytic activity relative to FhCL3 [102], it can still cleave collagen, and therefore, this enzyme likely assists migration throughout the host’s liver and into the bile ducts [104].

FhCL1, FhCL2, and FhCL5 can also digest host hemoglobin, suggesting they also have a key role in nutrient acquisition while the parasite is in the bile duct, where it is considered an obligate blood feeder [101, 105, 106]. The function of FhCL1 has been suggested to be primarily adapted to hemoglobin digestion [101]. The enzyme is the most predominant protease secreted by the adult fluke; it is liberated as a 37 kDa zymogen from the cecal epithelial cells lining the gut wall into the gut lumen, where it is activated to its 25 kDa mature form [101, 106]. In contrast to FhCL3, the FhCL1 S2 subsite exhibits a marked preference for hydrophobic amino acids such as Phe, Leu, and Ala, which are the most common residues found in hemoglobin. The low pH of the parasite gut is important in facilitating the unraveling of the hemoglobin to allow FhCL1, which is active at both acid and neutral pH, to access and cleave peptide bonds [106].

The various FhCLs also cleave IgG and can prevent antibody-dependent cell cytotoxicity [107]. Furthermore, FhCL1 alters macrophage function via the cleavage of Toll-like Receptor 3 (TLR3) within endosomes; accordingly, the proteases have been proposed to aid the modulation or impairment of host immune responses as the parasite migrates through the tissues [107, 108].

Cathepsin B cysteine proteases are also important in the interaction of *F*. *hepatica* with its host [104]. Similar to FhCL3, FhB1, FhB2, and FhB3 are highly expressed in and secreted from NEJs but are then down-regulated once the parasite penetrates the liver [5], which would suggest specific functions in tissue penetration and feeding [109]. Other members of the FhCB family are not secreted and, consequently, may play housekeeping functions within the parasite’s tissues. Finally, a *F*. *hepatica* cathepsin C dipeptidase, which removes dipeptides from the N-terminus of proteins, may further process peptides derived from the action of FhCL and FhCB proteases [101].

The importance of *F*. *hepatica* cysteine proteases in the invasion of tissues, the acquisition of nutrients, and in immunomodulation has made them key targets for the development of novel vaccines and drugs [104]. Indeed, vaccine studies in ruminants have demonstrated that recombinantly produced FhCLs offer substantial protection against experimental and field infections of liver flukes (reviewed in [104]).

## Cestoda

In contrast to trematodes, cestode cysteine proteases have received relatively little attention, and some ambiguity remains regarding their precise functions. Nonetheless, attempts to functionally characterize the proteases identified have been made. For example, *Taenia solium* and *T*. *pisiformis* express cysteine proteases (particularly cathepsin L) that cleave human IgG [110–112]. Also, cysteine proteases in the ESP of *T*. *solium* were found to deplete CD4+ T cells and induce apoptosis in vitro [113, 114]. Although the degradation of immune-related proteins suggests a role in parasite defense against the host immune system, the degradation of these proteins could simultaneously provide a nutritional benefit to the parasite [115], particularly as IgG proteins have been identified in the cysts of various *Taenia* species [111, 116, 117]. Indeed, the uptake and proteolysis of IgG (and other serum proteins) by the cyst has been observed [116]. Perhaps the degradation of IgG not only protects the parasite from recognition by the host immune system but also provides a supply of amino acids to sustain itself within host tissues [115–117].

Recombinant cathepsin L from *T*. *pisiformis* degrades fibronectin and thus may aid the degradation of host tissues and consequently invasion [112]. Given the similarity of this cathepsin L to orthologous proteases in *C*. *sinensis*, *F*. *hepatica*, and *P*. *westermani* [110], further investigation is warranted to more fully understand its possible contribution to the degradation of host proteins. Similarly, a cathepsin L and two cathepsins B from *Echinococcus multilocularis* were found to degrade IgG, albumin, collagen, and fibronectin [118, 119]. Therefore, as with the *Taenia* species enzymes, the *E*. *multilocularis* cysteine proteases may be involved in nutrient acquisition, immunomodulation, and tissue invasion [118, 119] and, accordingly, may prove to be useful drug targets.

## Planaria and *C*. *elegans* as models for cysteine protease activity

Because of the genetic intractability of most helminth parasites, it is difficult to characterize or interrogate protein function. Building the necessary tools, even those as basic as isolated marker mutations or genetic balancers, can take years, hindering the advancement of parasite research. One useful tool, transient RNAi, is variably effective in flatworm [120, 121] and roundworm parasites [122–124]. RNAi protocols differ widely but typically involve feeding, soaking, or electroporation with double-stranded RNA (dsRNA). Of these, electroporation is expensive and labor intensive and can cause parasite damage, e.g., to the larvae of *Brugia malayi* [125]. Furthermore, the ability to generate transgenic animals is dependent on parasite culture [126]. Long and complex life cycles involving more than one host may make the challenge of gene knockouts insurmountable for many parasites.

The use of free-living helminths to model parasitic nematodes and flatworms may compensate for many of these problems. *C*. *elegans* is a well-studied nematode with access to a great number of genetic tools. Transgenesis is routine, and transgenic animals can be easily enriched due to the ease of *C*. *elegans* culture. In addition, gene expression and protein localization can be observed with fluorescent reporters. Finally, gene editing using the CRISPR/Cas9 system is now available for *C*. *elegans* [126]. *C*. *elegans* has also been used as an expression system for parasite proteins [127]. Expression of recombinant proteins can be used in vaccination studies, as these proteins are properly glycosylated and activated in the nematode. Because of the structural, developmental, and reproductive similarities between *C*. *elegans* and parasitic nematodes, the tools available to study *C*. *elegans* can be exploited to find new targets that may be important in parasites. Although *C*. *elegans* is likely a more effective model for clade V nematodes rather than either clades III or IV [128], there is still a high degree of similarity between these clades, so findings with *C*. *elegans* should not be discounted.

Although parasitism evolved independently multiple times in nematodes [129], all parasitic flatworms are derived from the monophyletic group “neodermata,” encompassing some 6,000 species [130]. Therefore, the possibility exists that a free-living flatworm can serve as a good model for many parasitic flatworms. The planarian flatworm, *Schmidtea mediterranea*, shares many important features with parasitic flatworms, especially *Schistosoma*. Both lay ectolecithal eggs [130], possess structural similarities in their protonephridia [131, 132], and contain a population of stem cells called neoblasts that are distributed throughout the body [133]. These neoblasts have been the source of most research on *S*. *mediterranea*, and their regenerative capabilities makes the propagation of *Schmidtea* fast, cheap, and easy. *S*. *mediterranea* also has a relatively small diploid genome that is fully sequenced and assembled [134]. Both large-scale whole-mount in situ hybridization (WISH) and RNAi can be performed with relative ease [135, 136], thereby making new and interesting targets much easier to find, in contrast to parasitic flatworms. Many of the genetic tools available for *C*. *elegans* are still underdeveloped for *S*. *mediterranea*, but planarian worms are among the best-characterized and studied flatworms and can serve as a potential model system for the less-tractable parasites.

Even though both *C*. *elegans* and *S*. *mediterranea* have been valuable in the study of nematode and flatworm biology, proteases involved in protein digestion remain a relatively understudied area for both worms. For *C*. *elegans*, the biology of digestion has been categorized as “a blind spot in this otherwise remarkably well-characterized organism” [137]. Five cathepsin B-like genes have been identified as gut specific, although knockouts of each of these, or all five simultaneously, did not yield a phenotype [138]. This suggests that although cathepsin B proteases may be involved in digestion, they are not essential. Other cysteine cathepsins have been identified in *C*. *elegans*, although they appear not to contribute to digestion in the gut. RNAi of a cathepsin L is almost 100% embryonic lethal due to its role in degrading yolk during development [139], whereas RNAi of a cathepsin Z-like protease causes severe molting defects [140]. Thus, research on protease function in *C*. *elegans*, while still underdeveloped, could provide new insights, especially for orthologous proteases found in parasitic nematodes.

Very little is known about the functions of proteases in *S*. *mediterranea*. Recently, cysteine proteases, along with aspartic proteases, have been identified as the major participants in protein digestion in the *S*. *mediterranea* gut [141]. These cysteine proteases include several cathepsins B and L. One specific cathepsin B protease, SmedCB, has been characterized, and its contribution to protein digestion seems to be redundant with cathepsin L proteases. The identification and importance of these two protease types in planarian digestion is similar to findings for parasitic flatworms, including schistosomes and *Fasciola*. As RNAi in *S*. *mediterranea* is highly efficient, this free-living worm could be used to characterize cysteine proteases that are difficult to knock down and study in parasitic flatworms. Finally, because parasite proteases are diverse and exhibit varying degrees of specialization that may not be directly relatable to or interrogable in free-living worm models, the use of worm models to functionally characterize parasite proteases may be restricted to those molecules that are more conserved in function.

## Helminth cysteine proteases as drug targets: Proof-of-concept studies in small-animal models of schistosome and hookworm infection

Early work by Wasilewski and colleagues [76] demonstrated the potential utility of helminth cysteine proteases as drug targets. Specifically, they showed that daily intraperitoneal (i.p.) administration of approximately 100 mg/kg Mu-F-hF-FMK, a fluoromethyl ketone cysteine protease inhibitor, to mice harboring lung-stage or adult *S*. *mansoni* decreased worm burdens by 85% and 28%, respectively. The associated hepatic egg counts were decreased by 87% and 80%, respectively. This study was followed up 10 years later with the demonstration that the vinyl sulfone inhibitor, N-Me-piperazine-F-hF-vinyl sulfone phenyl (K11777), a preclinical candidate for Chagas disease (see the relevant chapter in this collection), could ameliorate a *S*. *mansoni* infection in mice. Specifically, when administered at 25 mg/kg twice daily i.p. for two weeks to target skin- and lung-migratory schistosome parasites, a >88% reduction in worm burden was achieved [74]. When administered for one week to mice harboring mature infections, worm burdens were reduced by 81% [74]. In both administration scenarios, the decrease in worm burdens was associated with pronounced egg burden reductions and associated liver and spleen pathologies. Importantly, in terms of the putative molecular target, the antiparasitic effect was correlated with a 90% decrease in the cathepsin B (SmCB1) activity of the parasite.

To pursue a target-based drug development program, crystal structures of SmCB1, bound to various small-molecule cysteine protease inhibitors, were generated [75]. Interestingly, the potency of inhibition of the recombinant SmCB1 [56] by these and other small-molecule inhibitors correlated with severity of the parasite’s (schistosomula) response phenotype in vitro [75]. Other strategies for SmCB1 inhibitor development have involved the use of small molecules derived from the propeptide (part of the SmCB1 zymogen that is removed during activation of the enzyme) [142] and a quantum mechanics scoring methodology to design and evaluate new inhibitors [143]. As attractive a target as SmCB1 is, it isn’t yet clear whether short courses of protease inhibitors would be significantly antiparasitic for eventual clinical use. This is in the context of the target product profile for new antischistosomal drugs that demands short-course (preferably single-dose) therapy [144] in order to facilitate mass drug administration (MDA), as is practiced for the current drug, praziquantel [145].

K11777 found a second life in a different animal model of helminth infection, namely the Golden Syrian hamster, which is a competent host for the hookworm *A*. *ceylanicum*. A single oral dose of 100 mg/kg provided near cure of infection [146]. Similar to the experiments with schistosomes, resident cathepsin B activity in the hookworm gut was decreased by over 95%. These efficacy and target-validation studies for K11777 and hookworm infection were supported by the use of a second and structurally unrelated cathepsin K inhibitor, namely, odanacatib (ODN) [147], which was withdrawn by Merck as a drug candidate for treatment of osteoporosis in September 2016. Specifically, a single oral dose of 100 mg/kg ODN decreased adult *A*. *ceylanicum* burdens by 73%, with a 51% reduction in the parasite’s cysteine protease activity. The efficacy measured was unexpected given ODN’s exquisite specificity for cathepsin K over other cathepsins, including cathepsin B [148, 149], and it was suggested that ODN’s long plasma half-life was key to the pronounced efficacy measured. Accordingly, it was reasoned that combining K11777’s potency for cathepsin B with ODN’s excellent pharmacokinetic properties could lead to an inhibitor that is efficacious at a lower dose [147].

As with MDA for treatment of schistosomiasis, single oral dosing is the modus operandi of MDA programs that deliver essential antinematodal drugs [150]. In this context, the activities of K11777 and ODN in the hamster model of hookworm infection are encouraging, and the search continues for cysteine protease inhibitors with improved on-target antiparasite efficacy at lower overall doses. Of importance to the success of any protease-inhibitor drug, however, will be an understanding of whether a substantial therapeutic benefit against the other, and often coendemic, soil-transmitted nematodes, namely, *Trichuris* and *Ascaris*, is also delivered.

**Key Learning Points**

1. Cysteine proteases are key contributors to the digestion of host proteins by helminth parasites.
2. Helminth cysteine proteases are often expressed as multigene families.
3. Helminth cysteine proteases as drug and vaccine targets is an active area of research.

**Top Five Papers**

1. Kasny M, Mikes L, Hampl V, Dvořák J, Caffrey CR, Dalton JP, et al. Chapter 4. Peptidases of trematodes. Adv Parasitol. 2009;69:205–97. Epub 2009/07/23. doi: S0065-308X(09)69004-7 [pii] 10.1016/S0065-308X(09)69004-7. PubMed PMID: 19622410.
2. Ranjit N, Zhan B, Stenzel DJ, Mulvenna J, Fujiwara R, Hotez PJ, et al. A family of cathepsin B cysteine proteases expressed in the gut of the human hookworm, *Necator americanus*. Mol Biochem Parasitol. 2008;160(2):90–9. Epub 2008/05/27. doi: S0166-6851(08)00094-7 [pii] 10.1016/j.molbiopara.2008.04.008. PubMed PMID: 18501979.
3. Cwiklinski K, Dalton JP, Dufresne PJ, La Course J, Williams DJ, Hodgkinson J, et al. The *Fasciola hepatica* genome: gene duplication and polymorphism reveals adaptation to the host environment and the capacity for rapid evolution. Genome Biol. 2015;16:71. Epub 2015/04/19. doi: 10.1186/s13059-015-0632-2 10.1186/s13059-015-0632-2 [pii]. PubMed PMID: 25887684.
4. Molina-Hernandez V, Mulcahy G, Perez J, Martinez-Moreno A, Donnelly S, O’Neill SM, et al. *Fasciola hepatica* vaccine: we may not be there yet but we’re on the right road. Vet Parasitol. 2015;208(1–2):101–11. Epub 2015/02/07. doi: S0304-4017(15)00008-4 [pii] 10.1016/j.vetpar.2015.01.004. PubMed PMID: 25657086.
5. Vermeire JJ, Lantz LD, Caffrey CR. Cure of hookworm infection with a cysteine protease inhibitor. PLoS Negl Trop Dis. 2012;6(7):e1680. Epub 2012/07/18. doi: 10.1371/journal.pntd.0001680 PNTD-D-12-00199 [pii]. PubMed PMID: 22802972.

## References

[1] Caffrey CR, McKerrow JH, Salter JP, Sajid M. Blood ‘n’ guts: an update on schistosome digestive peptidases. Trends Parasitol. 2004;20(5):241–8. Epub 2004/04/24. 10.1016/j.pt.2004.03.004 . 15105025

[2] Caffrey C, Britton C, McKerrow JH. Helminth cysteine proteases Handbook of Proteolytic Enzymes. 3rd ed Oxford: Elsevier; 2011.

[3] Knox D. Proteases in blood-feeding nematodes and their potential as vaccine candidates. Adv Exp Med Biol. 2011;712:155–76. Epub 2011/06/11. 10.1007/978-1-4419-8414-2_10 . 21660664

[4] Ranjit N, Zhan B, Stenzel DJ, Mulvenna J, Fujiwara R, Hotez PJ, et al A family of cathepsin B cysteine proteases expressed in the gut of the human hookworm, *Necator americanus*. Mol Biochem Parasitol. 2008;160(2):90–9. Epub 2008/05/27. 10.1016/j.molbiopara.2008.04.008 . 18501979

[5] Cwiklinski K, Dalton JP, Dufresne PJ, La Course J, Williams DJ, Hodgkinson J, et al The *Fasciola hepatica* genome: gene duplication and polymorphism reveals adaptation to the host environment and the capacity for rapid evolution. Genome Biol. 2015;16:71 Epub 2015/04/19. 10.1186/s13059-015-0632-2 . 25887684 PMC4404566

[6] Jasmer DP, Mitreva MD, McCarter JP. mRNA sequences for *Haemonchus contortus* intestinal cathepsin B-like cysteine proteases display an extreme in abundance and diversity compared with other adult mammalian parasitic nematodes. Mol Biochem Parasitol. 2004;137(2):297–305. Epub 2004/09/24. 10.1016/j.molbiopara.2004.06.010 . 15383300

[7] Caffrey CR, editor. Parasitic Helminths: Targets, Screens, Drugs and Vaccines. Weinheim: Wiley-Blackwell; 2012.

[8] Morera P, Cespedes R. *Angiostrongylus costaricensis* n. sp. (Nematoda: Metastrongyloidea), a new lungworm occurring in man in Costa Rica. Rev Biol Trop. 1970;18(1):173–85. Epub 1970/07/01. . 5527668

[9] Chen H. Un nouveau nématode pulmonaire, *Pulmonema cantonensis* ng, n. sp., des rats de Canton. Ann Parasitol Hum Comp. 1935;13(2):312–4.

[10] Han YP, Li ZY, Li BC, Sun X, Zhu CC, Ling XT, et al Molecular cloning and characterization of a cathepsin B from *Angiostrongylus cantonensis*. Parasitol Res. 2011;109(2):369–78. Epub 2011/02/24. 10.1007/s00436-011-2264-0 . 21344211

[11] Long Y, Cao B, Yu L, Tukayo M, Feng C, Wang Y, et al *Angiostrongylus cantonensis* cathepsin B-like protease (Ac-cathB-1) is involved in host gut penetration. Parasite. 2015;22:37 Epub 2015/12/20. 10.1051/parasite/2015037 . 26682577 PMC4684300

[12] Ni F, Wang Y, Zhang J, Yu L, Fang W, Luo D. Cathepsin B-like and hemoglobin-type cysteine proteases: stage-specific gene expression in *Angiostrongylus cantonensis*. Exp Parasitol. 2012;131(4):433–41. Epub 2012/06/07. 10.1016/j.exppara.2012.05.014 . 22668746

[13] Morassutti AL, Levert K, Pinto PM, da Silva AJ, Wilkins P, Graeff-Teixeira C. Characterization of *Angiostrongylus cantonensis* excretory-secretory proteins as potential diagnostic targets. Exp Parasitol. 2012;130(1):26–31. Epub 2011/10/25. 10.1016/j.exppara.2011.10.003 . 22019415

[14] Cheng M, Yang X, Li Z, He H, Qu Z, He A, et al Cloning and characterization of a novel cathepsin B-like cysteine proteinase from *Angiostrongylus cantonensis*. Parasitol Res. 2012;110(6):2413–22. Epub 2012/01/05. 10.1007/s00436-011-2780-y . 22215189

[15] Yu C, Wang Y, Zhang J, Fang W, Luo D. Immunolocalization and developmental expression patterns of two cathepsin B proteases (AC-cathB-1, -2) of *Angiostrongylus cantonensis*. Exp Parasitol. 2014;144:27–33. Epub 2014/06/15. 10.1016/j.exppara.2014.06.008 . 24929149

[16] Anten JF, Zuidema PJ. Hookworm infection in Dutch servicemen returning from West New Guinea. Trop Geogr Med. 1964;64(756):216–24. Epub 1964/09/01. . 5895548

[17] Chowdhury AB, Schad GA. *Ancylostoma ceylanicum*: a parasite of man in Calcutta and environs. Am J Trop Med Hyg. 1972;21(3):300–1. Epub 1972/05/01. . 5025614 10.4269/ajtmh.1972.21.300

[18] Lee DL. Life Cycles In: Lee DL, editor. The Biology of Nematodes. London: Taylor & Francis; 2002 p. 141–62.

[19] Despommier DD, Gwadz R.W., Hotez P.J., Knirsch C.A. Parasitic Diseases. 5th ed New York: Apple Trees productions LLC; 2006.

[20] Ranjit N, Jones MK, Stenzel DJ, Gasser RB, Loukas A. A survey of the intestinal transcriptomes of the hookworms, *Necator americanus* and *Ancylostoma caninum*, using tissues isolated by laser microdissection microscopy. Int J Parasitol. 2006;36(6):701–10. Epub 2006/03/21. 10.1016/j.ijpara.2006.01.015 . 16545815

[21] Jasmer DP, Roth J, Myler PJ. Cathepsin B-like cysteine proteases and *Caenorhabditis elegans* homologues dominate gene products expressed in adult *Haemonchus contortus* intestine. Mol Biochem Parasitol. 2001;116(2):159–69. . 11522349 10.1016/s0166-6851(01)00312-7

[22] Wei J, Damania A, Gao X, Liu Z, Mejia R, Mitreva M, et al The hookworm *Ancylostoma ceylanicum* intestinal transcriptome provides a platform for selecting drug and vaccine candidates. Parasit Vectors. 2016;9(1):518 10.1186/s13071-016-1795-8 . 27677574 PMC5039805

[23] Ranjit N, Zhan B, Hamilton B, Stenzel D, Lowther J, Pearson M, et al Proteolytic degradation of hemoglobin in the intestine of the human hookworm *Necator americanus*. J Infect Dis. 2009;199(6):904–12. Epub 2009/05/13. . 19434933 10.1086/597048

[24] Pearson MS, Ranjit N, Loukas A. Blunting the knife: development of vaccines targeting digestive proteases of blood-feeding helminth parasites. Biol Chem. 2010;391(8):901–11. Epub 2010/05/21. 10.1515/BC.2010.074 . 20482309

[25] Dowd AJ, Dalton JP, Loukas AC, Prociv P, Brindley PJ. Secretion of cysteine proteinase activity by the zoonotic hookworm Ancylostoma caninum. Am J Trop Med Hyg. 1994;51(3):341–7. Epub 1994/09/01. . 7943555 10.4269/ajtmh.1994.51.341

[26] Harrop SA, Sawangjaroen N, Prociv P, Brindley PJ. Characterization and localization of cathepsin B proteinases expressed by adult *Ancylostoma caninum* hookworms. Mol Biochem Parasitol. 1995;71(2):163–71. Epub 1995/05/01. . 7477098 10.1016/0166-6851(95)00045-3

[27] Mieszczanek J, Kofta W, Wedrychowicz H. Molecular cloning of a cysteine proteinase cDNA from adult *Ancylostoma ceylanicum* by the method of rapid amplification of cDNA ends using polymerase chain reaction. Parasitol Res. 2000;86(12):993–8. Epub 2001/01/02. . 11133115 10.1007/pl00008531

[28] Tang YT, Gao X, Rosa BA, Abubucker S, Hallsworth-Pepin K, Martin J, et al Genome of the human hookworm *Necator americanus*. Nat Genet. 2014 Epub 2014/01/21. 10.1038/ng.2875 . 24441737 PMC3978129

[29] Yatsuda AP, Bakker N, Krijgsveld J, Knox DP, Heck AJ, de Vries E. Identification of secreted cysteine proteases from the parasitic nematode *Haemonchus contortus* detected by biotinylated inhibitors. Infect Immun. 2006;74(3):1989–93. Epub 2006/02/24. 10.1128/IAI.74.3.1989-1993.2006 . 16495580 PMC1418636

[30] Karanu FN, Rurangirwa FR, McGuire TC, Jasmer DP. *Haemonchus contortus*: inter- and intrageographic isolate heterogeneity of proteases in adult worm excretory-secretory products. Exp Parasitol. 1997;86(1):89–91. Epub 1997/05/01. 10.1006/expr.1997.4158 . 9149245

[31] Skuce PJ, Redmond DL, Liddell S, Stewart EM, Newlands GF, Smith WD, et al Molecular cloning and characterization of gut-derived cysteine proteinases associated with a host protective extract from *Haemonchus contortus*. Parasitology. 1999;119(Pt 4):405–12. Epub 1999/12/03. . 10581619 10.1017/s0031182099004813

[32] Allaie IM, Prasad A, Sankar M. Cysteine proteinase genes in Indian strain of *Haemonchus contortus*. Mol Biochem Parasitol. 2014;196(2):117–21. Epub 2014/09/23. 10.1016/j.molbiopara.2014.09.003 . 25239651

[33] Martin S, Molina JM, Hernandez YI, Ferrer O, Munoz MC, Lopez A, et al Influence of immunoprotection on genetic variability of cysteine proteinases from *Haemonchus contortus* adult worms. Int J Parasitol. 2015;45(13):831–40. Epub 2015/08/05. 10.1016/j.ijpara.2015.06.006 . 26241655

[34] Rhoads ML, Fetterer RH. Developmentally regulated secretion of cathepsin L-like cysteine proteases by *Haemonchus contortus*. J Parasitol. 1995;81(4):505–12. Epub 1995/08/01. . 7623189

[35] Knox DP, Smith SK, Smith WD. Immunization with an affinity purified protein extract from the adult parasite protects lambs against infection with *Haemonchus contortus*. Parasite Immunol. 1999;21(4):201–10. Epub 1999/05/13. . 10320617 10.1046/j.1365-3024.1999.00220.x

[36] Ruiz A, Molina JM, Gonzalez JF, Conde MM, Martin S, Hernandez YI. Immunoprotection in goats against *Haemonchus contortus* after immunization with cysteine protease enriched protein fractions. Vet Res. 2004;35(5):565–72. Epub 2004/09/17. 10.1051/vetres:2004032 . 15369659

[37] Bethony J, Brooker S, Albonico M, Geiger SM, Loukas A, Diemert D, et al Soil-transmitted helminth infections: ascariasis, trichuriasis, and hookworm. Lancet. 2006;367(9521):1521–32. Epub 2006/05/09. 10.1016/S0140-6736(06)68653-4 . 16679166

[38] Betson M, Nejsum P, Bendall RP, Deb RM, Stothard JR. Molecular epidemiology of ascariasis: a global perspective on the transmission dynamics of *Ascaris* in people and pigs. J Infect Dis. 2014;210(6):932–41. Epub 2014/04/02. 10.1093/infdis/jiu193 . 24688073 PMC4136802

[39] Drake LJ, Bianco AE, Bundy DA, Ashall F. Characterization of peptidases of adult *Trichuris muris*. Parasitology. 1994;109(Pt 5):623–30. Epub 1994/12/01. . 7831097 10.1017/s0031182000076502

[40] Hill DE, Sakanari JA. *Trichuris suis*: thiol protease activity from adult worms. Exp Parasitol. 1997;85(1):55–62. Epub 1997/01/01. 10.1006/expr.1996.4125 . 9024202

[41] Hasnain SZ, McGuckin MA, Grencis RK, Thornton DJ. Serine protease(s) secreted by the nematode *Trichuris muris* degrade the mucus barrier. PLoS Negl Trop Dis. 2012;6(10):e1856 Epub 2012/10/17. 10.1371/journal.pntd.0001856 . 23071854 PMC3469553

[42] Foth BJ, Tsai IJ, Reid AJ, Bancroft AJ, Nichol S, Tracey A, et al Whipworm genome and dual-species transcriptome analyses provide molecular insights into an intimate host-parasite interaction. Nat Genet. 2014;46(7):693–700. Epub 2014/06/16. 10.1038/ng.3010 . 24929830 PMC5012510

[43] Santos LN, Silva ES, Santos AS, De Sa PH, Ramos RT, Silva A, et al De novo assembly and characterization of the *Trichuris trichiura* adult worm transcriptome using Ion Torrent sequencing. Acta Trop. 2016;159:132–41. Epub 2016/04/04. 10.1016/j.actatropica.2016.03.036 . 27038556

[44] Santos LN, Gallo MB, Silva ES, Figueiredo CA, Cooper PJ, Barreto ML, et al A proteomic approach to identify proteins from *Trichuris trichiura* extract with immunomodulatory effects. Parasite Immunol. 2013;35(5–6):188–93. Epub 2013/02/13. 10.1111/pim.12025 . 23398517 PMC7612816

[45] Maki J, Furuhashi A, Yanagisawa T. The activity of acid proteases hydrolysing haemoglobin in parasitic helminths with special reference to interspecific and intraspecific distribution. Parasitology. 1982;84(1):137–47. Epub 1982/02/01. . 7038595 10.1017/s0031182000051738

[46] Maki J, Yanagisawa T. Demonstration of carboxyl and thiol protease activities in adult *Schistosoma mansoni*, *Dirofilaria immitis*, *Angiostrongylus cantonensis* and *Ascaris suum*. J Helminthol. 1986;60(1):31–7. Epub 1986/03/01. . 3517135 10.1017/s0022149x00008191

[47] Jasmer DP, Rosa BA, Mitreva M. Peptidases compartmentalized to the *Ascaris suum* intestinal lumen and apical intestinal membrane. PLoS Negl Trop Dis. 2015;9(1):e3375 Epub 2015/01/09. 10.1371/journal.pntd.0003375 . 25569475 PMC4287503

[48] Jex AR, Liu S, Li B, Young ND, Hall RS, Li Y, et al *Ascaris suum* draft genome. Nature. 2011;479(7374):529–33. Epub 2011/10/28. 10.1038/nature10553 . 22031327

[49] Cheever AW, Kamel IA, Elwi AM, Mosimann JE, Danner R, Sippel JE. *Schistosoma mansoni* and *S*. *haematobium* infections in Egypt. III. Extrahepatic pathology. Am J Trop Med Hyg. 1978;27(1 Pt 1):55–75. Epub 1978/01/01. . 626283 10.4269/ajtmh.1978.27.55

[50] Timms AR, Bueding E. Studies of a proteolytic enzyme from *Schistosoma mansoni*. Br J Pharmacol Chemother. 1959;14(1):68–73. Epub 1959/03/01. . 13651581 10.1111/j.1476-5381.1959.tb00930.x PMC1481838

[51] Kasny M, Mikes L, Hampl V, Dvořák J, Caffrey CR, Dalton JP, et al Chapter 4. Peptidases of trematodes. Adv Parasitol. 2009;69:205–97. Epub 2009/07/23. 10.1016/S0065-308X(09)69004-7 . 19622410

[52] Dvořák J, Mashiyama ST, Sajid M, Braschi S, Delcroix M, Schneider EL, et al SmCL3, a gastrodermal cysteine protease of the human blood fluke *Schistosoma mansoni*. PLoS Negl Trop Dis. 2009;3(6):e449 Epub 2009/06/03. 10.1371/journal.pntd.0000449 . 19488406 PMC2685030

[53] Dalton JP, Brindley PJ, Donnelly S, Robinson MW. The enigmatic asparaginyl endopeptidase of helminth parasites. Trends Parasitol. 2009;25(2):59–61. Epub 2008/12/23. 10.1016/j.pt.2008.11.002 . 19101207

[54] El-Sayed LH, Ghoneim H, Demian SR, El-Sayed MH, Tawfik NM, Sakr I, et al Diagnostic significance of *Schistosoma mansoni* proteins Sm31 and Sm32 in human schistosomiasis in an endemic area in Egypt. Trop Med Int Health. 1998;3(9):721–7. Epub 1998/10/01. . 9754667 10.1046/j.1365-3156.1998.00298.x

[55] Noya O, De Noya BA, Ballen DE, Bermudez H, Bout D, Hoebeke J. Immunogenicity of synthetic peptides from the Sm31 antigen (cathepsin B) of the *Schistosoma mansoni* adult worms. Parasite Immunol. 2001;23(11):567–73. Epub 2001/11/13. . 11703807 10.1046/j.1365-3024.2001.00414.x

[56] Sajid M, McKerrow JH, Hansell E, Mathieu MA, Lucas KD, Hsieh I, et al Functional expression and characterization of *Schistosoma mansoni* cathepsin B and its trans-activation by an endogenous asparaginyl endopeptidase. Mol Biochem Parasitol. 2003;131(1):65–75. Epub 2003/09/12. . 12967713 10.1016/s0166-6851(03)00194-4

[57] Sulbaran GS, Ballen DE, Bermudez H, Lorenzo M, Noya O, Cesari IM. Detection of the Sm31 antigen in sera of *Schistosoma mansoni*- infected patients from a low endemic area. Parasite Immunol. 2010;32(1):20–8. Epub 2010/01/01. 10.1111/j.1365-3024.2009.01152.x . 20042004

[58] El Ridi R, Tallima H, Selim S, Donnelly S, Cotton S, Gonzales Santana B, et al Cysteine peptidases as schistosomiasis vaccines with inbuilt adjuvanticity. PLoS ONE. 2014;9(1):e85401 Epub 2014/01/28. 10.1371/journal.pone.0085401 . 24465551 PMC3897446

[59] Gonzalez AY, Sulbaran GS, Ballen DE, Cesari IM. Immunocapture of circulating *Schistosoma mansoni* cathepsin B antigen (Sm31) by anti-Sm31 polyclonal antibodies. Parasitol Int. 2016;65(3):191–5. Epub 2015/12/29. 10.1016/j.parint.2015.12.008 . 26709076

[60] Ruppel A, Diesfeld HJ, Rother U. Immunoblot analysis of *Schistosoma mansoni* antigens with sera of schistosomiasis patients: diagnostic potential of an adult schistosome polypeptide. Clin Exp Immunol. 1985;62(3):499–506. Epub 1985/12/01. . 3936655 PMC1577465

[61] Klinkert MQ, Felleisen R, Link G, Ruppel A, Beck E. Primary structures of Sm31/32 diagnostic proteins of *Schistosoma mansoni* and their identification as proteases. Mol Biochem Parasitol. 1989;33(2):113–22. Epub 1989/03/01. . 2725581 10.1016/0166-6851(89)90025-x

[62] Smith AM, Dalton JP, Clough KA, Kilbane CL, Harrop SA, Hole N, et al Adult *Schistosoma mansoni* express cathepsin L proteinase activity. Mol Biochem Parasitol. 1994;67(1):11–9. Epub 1994/09/01. . 7838171 10.1016/0166-6851(94)90091-4

[63] Brady CP, Dowd AJ, Brindley PJ, Ryan T, Day SR, Dalton JP. Recombinant expression and localization of *Schistosoma mansoni* cathepsin L1 support its role in the degradation of host hemoglobin. Infect Immun. 1999;67(1):368–74. Epub 1998/12/24. . 9864238 10.1128/iai.67.1.368-374.1999 PMC96319

[64] Day SR, Dalton JP, Clough KA, Leonardo L, Tiu WU, Brindley PJ. Characterization and cloning of the cathepsin L proteinases of *Schistosoma japonicum*. Biochem Biophys Res Commun. 1995;217(1):1–9. Epub 1995/12/05. . 8526895 10.1006/bbrc.1995.2737

[65] Michel A, Ghoneim H, Resto M, Klinkert MQ, Kunz W. Sequence, characterization and localization of a cysteine proteinase cathepsin L in *Schistosoma mansoni*. Mol Biochem Parasitol. 1995;73(1–2):7–18. Epub 1995/07/01. . 8577349 10.1016/0166-6851(95)00092-f

[66] Bogitsh BJ, Dalton JP, Brady CP, Brindley PJ. Gut-associated immunolocalization of the *Schistosoma mansoni* cysteine proteases, SmCL1 and SmCL2. J Parasitol. 2001;87(2):237–41. Epub 2001/04/25. 10.1645/0022-3395(2001)087[0237:GAIOTS]2.0.CO;2 . 11318550

[67] Delcroix M, Sajid M, Caffrey CR, Lim KC, Dvořák J, Hsieh I, et al A multienzyme network functions in intestinal protein digestion by a platyhelminth parasite. J Biol Chem. 2006;281(51):39316–29. Epub 2006/10/10. 10.1074/jbc.M607128200 . 17028179

[68] Dalton JP, Hola-Jamriska L, Brindley PJ. Asparaginyl endopeptidase activity in adult *Schistosoma mansoni*. Parasitology. 1995;111(Pt 5):575–80. Epub 1995/12/01. . 8559590 10.1017/s0031182000077052

[69] Krautz-Peterson G, Skelly PJ. Schistosome asparaginyl endopeptidase (legumain) is not essential for cathepsin B1 activation in vivo. Mol Biochem Parasitol. 2008;159(1):54–8. Epub 2008/02/19. 10.1016/j.molbiopara.2007.12.011 . 18280591 PMC2396453

[70] Jilkova A, Horn M, Rezacova P, Maresova L, Fajtova P, Brynda J, et al Activation route of the *Schistosoma mansoni* cathepsin B1 drug target: structural map with a glycosaminoglycan switch. Structure. 2014;22(12):1786–98. Epub 2014/12/03. 10.1016/j.str.2014.09.015 . 25456815

[71] Hola-Jamriska L, King LT, Dalton JP, Mann VH, Aaskov JG, Brindley PJ. Functional expression of dipeptidyl peptidase I (Cathepsin C) of the oriental blood fluke *Schistosoma japonicum* in Trichoplusia ni insect cells. Protein Expr Purif. 2000;19(3):384–92. Epub 2000/07/27. 10.1006/prep.2000.1261 . 10910729

[72] Hola-Jamriska L, Dalton JP, Aaskov J, Brindley PJ. Dipeptidyl peptidase I and III activities of adult schistosomes. Parasitology. 1999;118(Pt 3):275–82. Epub 1999/04/17. . 10205803 10.1017/s0031182098003746

[73] McCarthy E, Stack C, Donnelly SM, Doyle S, Mann VH, Brindley PJ, et al Leucine aminopeptidase of the human blood flukes, *Schistosoma mansoni* and *Schistosoma japonicum*. Int J Parasitol. 2004;34(6):703–14. Epub 2004/04/28. 10.1016/j.ijpara.2004.01.008 . 15111092

[74] Abdulla MH, Lim KC, Sajid M, McKerrow JH, Caffrey CR. *Schistosomiasis mansoni*: novel chemotherapy using a cysteine protease inhibitor. PLoS Med. 2007;4(1):e14 Epub 2007/01/12. 10.1371/journal.pmed.0040014 . 17214506 PMC1764436

[75] Jilkova A, Rezacova P, Lepsik M, Horn M, Vachova J, Fanfrlik J, et al Structural basis for inhibition of cathepsin B drug target from the human blood fluke, *Schistosoma mansoni*. J Biol Chem. 2011;286(41):35770–81. Epub 2011/08/13. 10.1074/jbc.M111.271304 . 21832058 PMC3195637

[76] Wasilewski MM, Lim KC, Phillips J, McKerrow JH. Cysteine protease inhibitors block schistosome hemoglobin degradation in vitro and decrease worm burden and egg production in vivo. Mol Biochem Parasitol. 1996;81(2):179–89. Epub 1996/10/30. . 8898333 10.1016/0166-6851(96)02703-x

[77] Ricciardi A, Visitsunthorn K, Dalton JP, Ndao M. A vaccine consisting of *Schistosoma mansoni* cathepsin B formulated in Montanide ISA 720 VG induces high level protection against murine schistosomiasis. BMC Infect Dis. 2016;16:112 Epub 2016/03/08. 10.1186/s12879-016-1444-z . 26945988 PMC4779570

[78] Horak P, Mikes L, Rudolfova J, Kolarova L. Penetration of *Trichobilharzia cercariae* into mammals: dangerous or negligible event? Parasite. 2008;15(3):299–303. Epub 2008/09/26. 10.1051/parasite/2008153299 . 18814698

[79] Doleckova K, Albrecht T, Mikes L, Horak P. Cathepsins B1 and B2 in the neuropathogenic schistosome *Trichobilharzia regenti*: distinct gene expression profiles and presumptive roles throughout the life cycle. Parasitol Res. 2010;107(3):751–5. Epub 2010/06/18. 10.1007/s00436-010-1943-6 . 20556428

[80] Kasny M, Mikes L, Doleckova K, Hampl V, Dvořák J, Novotny M, et al Cathepsins B1 and B2 of *Trichobilharzia* spp., bird schistosomes causing cercarial dermatitis. Adv Exp Med Biol. 2011;712:136–54. Epub 2011/06/11. 10.1007/978-1-4419-8414-2_9 . 21660663

[81] Dvořák J, Delcroix M, Rossi A, Vopalensky V, Pospisek M, Sedinova M, et al Multiple cathepsin B isoforms in schistosomula of *Trichobilharzia regenti*: identification, characterisation and putative role in migration and nutrition. Int J Parasitol. 2005;35(8):895–910. Epub 2005/06/14. 10.1016/j.ijpara.2005.02.018 . 15950230

[82] Chen W, Ning D, Wang X, Chen T, Lv X, Sun J, et al Identification and characterization of *Clonorchis sinensis* cathepsin B proteases in the pathogenesis of clonorchiasis. Parasit Vectors. 2015;8:647 Epub 2015/12/23. 10.1186/s13071-015-1248-9 . 26691339 PMC4687107

[83] Hong ST, Fang Y. *Clonorchis sinensis* and clonorchiasis, an update. Parasitol Int. 2012;61(1):17–24. Epub 2011/07/12. 10.1016/j.parint.2011.06.007 . 21741496

[84] Chen W, Wang X, Li X, Lv X, Zhou C, Deng C, et al Molecular characterization of cathepsin B from *Clonorchis sinensis* excretory/secretory products and assessment of its potential for serodiagnosis of clonorchiasis. Parasit Vectors. 2011;4:149 Epub 2011/07/29. 10.1186/1756-3305-4-149 . 21794140 PMC3163202

[85] Yoo WG, Kim DW, Ju JW, Cho PY, Kim TI, Cho SH, et al Developmental transcriptomic features of the carcinogenic liver fluke, *Clonorchis sinensis*. PLoS Negl Trop Dis. 2011;5(6):e1208 Epub 2011/07/09. 10.1371/journal.pntd.0001208 . 21738807 PMC3125140

[86] Kang JM, Bahk YY, Cho PY, Hong SJ, Kim TS, Sohn WM, et al A family of cathepsin F cysteine proteases of *Clonorchis sinensis* is the major secreted proteins that are expressed in the intestine of the parasite. Mol Biochem Parasitol. 2010;170(1):7–16. Epub 2009/11/26. 10.1016/j.molbiopara.2009.11.006 . 19932715

[87] Ju JW, Joo HN, Lee MR, Cho SH, Cheun HI, Kim JY, et al Identification of a serodiagnostic antigen, legumain, by immunoproteomic analysis of excretory-secretory products of *Clonorchis sinensis* adult worms. Proteomics. 2009;9(11):3066–78. Epub 2009/06/16. 10.1002/pmic.200700613 . 19526557

[88] Huang Y, Chen W, Wang X, Liu H, Chen Y, Guo L, et al The carcinogenic liver fluke, *Clonorchis sinensis*: new assembly, reannotation and analysis of the genome and characterization of tissue transcriptomes. PLoS ONE. 2013;8(1):e54732 Epub 2013/02/06. 10.1371/journal.pone.0054732 . 23382950 PMC3559784

[89] Zheng M, Hu K, Liu W, Hu X, Hu F, Huang L, et al Proteomic analysis of excretory secretory products from Clonorchis sinensis adult worms: molecular characterization and serological reactivity of a excretory-secretory antigen-fructose-1,6-bisphosphatase. Parasitol Res. 2011;109(3):737–44. Epub 2011/03/23. 10.1007/s00436-011-2316-5 . 21424807

[90] Li Y, Hu X, Liu X, Xu J, Hu F, Ma C, et al Molecular cloning and analysis of stage and tissue-specific expression of Cathepsin L-like protease from *Clonorchis sinensis*. Parasitol Res. 2009;105(2):447–52. Epub 2009/03/25. 10.1007/s00436-009-1406-0 . 19308452

[91] Nagano I, Pei F, Wu Z, Wu J, Cui H, Boonmars T, et al Molecular expression of a cysteine proteinase of *Clonorchis sinensis* and its application to an enzyme-linked immunosorbent assay for immunodiagnosis of clonorchiasis. Clin Diagn Lab Immunol. 2004;11(2):411–6. Epub 2004/03/12. 10.1128/CDLI.11.2.411-416.2004 . 15013996 PMC371220

[92] Lv X, Chen W, Wang X, Li X, Sun J, Deng C, et al Molecular characterization and expression of a cysteine protease from *Clonorchis sinensis* and its application for serodiagnosis of clonorchiasis. Parasitol Res. 2012;110(6):2211–9. Epub 2011/12/16. 10.1007/s00436-011-2751-3 . 22170263

[93] Na BK, Kang JM, Sohn WM. CsCF-6, a novel cathepsin F-like cysteine protease for nutrient uptake of *Clonorchis sinensis*. Int J Parasitol. 2008;38(5):493–502. Epub 2007/10/20. 10.1016/j.ijpara.2007.09.001 . 17945236

[94] Kang JM, Lee KH, Sohn WM, Na BK. Identification and functional characterization of CsStefin-1, a cysteine protease inhibitor of *Clonorchis sinensis*. Mol Biochem Parasitol. 2011;177(2):126–34. Epub 2011/03/01. 10.1016/j.molbiopara.2011.02.010 . 21354219

[95] Kang JM, Ju HL, Lee KH, Kim TS, Pak JH, Sohn WM, et al Identification and characterization of the second cysteine protease inhibitor of *Clonorchis sinensis* (CsStefin-2). Parasitol Res. 2014;113(1):47–58. Epub 2013/10/09. 10.1007/s00436-013-3624-8 . 24100605

[96] Blair D. Paragonimiasis. Adv Exp Med Biol. 2014;766:115–52. Epub 2014/06/07. 10.1007/978-1-4939-0915-5_5 . 24903365

[97] Lee EG, Na BK, Bae YA, Kim SH, Je EY, Ju JW, et al Identification of immunodominant excretory-secretory cysteine proteases of adult *Paragonimus westermani* by proteome analysis. Proteomics. 2006;6(4):1290–300. Epub 2006/01/13. 10.1002/pmic.200500399 . 16404718

[98] Na BK, Kim SH, Lee EG, Kim TS, Bae YA, Kang I, et al Critical roles for excretory-secretory cysteine proteases during tissue invasion of *Paragonimus westermani* newly excysted metacercariae. Cell Microbiol. 2006;8(6):1034–46. Epub 2006/05/10. 10.1111/j.1462-5822.2006.00685.x . 16681843

[99] McNulty SN, Fischer PU, Townsend RR, Curtis KC, Weil GJ, Mitreva M. Systems biology studies of adult paragonimus lung flukes facilitate the identification of immunodominant parasite antigens. PLoS Negl Trop Dis. 2014;8(10):e3242 Epub 2014/10/21. 10.1371/journal.pntd.0003242 . 25329661 PMC4199545

[100] Choi JH, Lee JH, Yu HS, Jeong HJ, Kim J, Hong YC, et al Molecular and biochemical characterization of hemoglobinase, a cysteine proteinase, in *Paragonimus westermani*. Korean J Parasitol. 2006;44(3):187–96. Epub 2006/09/14. 10.3347/kjp.2006.44.3.187 . 16969056 PMC2532661

[101] Robinson MW, Dalton JP, Donnelly S. Helminth pathogen cathepsin proteases: it’s a family affair. Trends Biochem Sci. 2008;33(12):601–8. Epub 2008/10/14. 10.1016/j.tibs.2008.09.001 . 18848453

[102] Robinson MW, Corvo I, Jones PM, George AM, Padula MP, To J, et al Collagenolytic activities of the major secreted cathepsin L peptidases involved in the virulence of the helminth pathogen, *Fasciola hepatica*. PLoS Negl Trop Dis. 2011;5(4):e1012 Epub 2011/04/13. 10.1371/journal.pntd.0001012 . 21483711 PMC3071364

[103] Corvo I, Cancela M, Cappetta M, Pi-Denis N, Tort JF, Roche L. The major cathepsin L secreted by the invasive juvenile *Fasciola hepatica* prefers proline in the S2 subsite and can cleave collagen. Mol Biochem Parasitol. 2009;167(1):41–7. Epub 2009/04/23. 10.1016/j.molbiopara.2009.04.005 . 19383516

[104] Molina-Hernandez V, Mulcahy G, Perez J, Martinez-Moreno A, Donnelly S, O’Neill SM, et al *Fasciola hepatica* vaccine: we may not be there yet but we’re on the right road. Vet Parasitol. 2015;208(1–2):101–11. Epub 2015/02/07. 10.1016/j.vetpar.2015.01.004 . 25657086 PMC4366043

[105] Norbury LJ, Beckham S, Pike RN, Grams R, Spithill TW, Fecondo JV, et al Adult and juvenile *Fasciola* cathepsin L proteases: different enzymes for different roles. Biochimie. 2011;93(3):604–11. Epub 2010/12/21. 10.1016/j.biochi.2010.12.004 . 21167899

[106] Lowther J, Robinson MW, Donnelly SM, Xu W, Stack CM, Matthews JM, et al The importance of pH in regulating the function of the *Fasciola hepatica* cathepsin L1 cysteine protease. PLoS Negl Trop Dis. 2009;3(1):e369 Epub 2009/01/28. 10.1371/journal.pntd.0000369 . 19172172 PMC2621350

[107] Berasain P, Carmona C, Frangione B, Dalton JP, Goni F. *Fasciola hepatica*: parasite-secreted proteinases degrade all human IgG subclasses: determination of the specific cleavage sites and identification of the immunoglobulin fragments produced. Exp Parasitol. 2000;94(2):99–110. Epub 2000/02/16. 10.1006/expr.1999.4479 . 10673346

[108] Donnelly S, O’Neill SM, Stack CM, Robinson MW, Turnbull L, Whitchurch C, et al Helminth cysteine proteases inhibit TRIF-dependent activation of macrophages via degradation of TLR3. J Biol Chem. 2010;285(5):3383–92. Epub 2009/11/20. 10.1074/jbc.M109.060368 . 19923225 PMC2823461

[109] McGonigle L, Mousley A, Marks NJ, Brennan GP, Dalton JP, Spithill TW, et al The silencing of cysteine proteases in *Fasciola hepatica* newly excysted juveniles using RNA interference reduces gut penetration. Int J Parasitol. 2008;38(2):149–55. Epub 2007/12/01. 10.1016/j.ijpara.2007.10.007 . 18048044

[110] Li AH, Moon SU, Park YK, Na BK, Hwang MG, Oh CM, et al Identification and characterization of a cathepsin L-like cysteine protease from *Taenia solium* metacestode. Vet Parasitol. 2006;141(3–4):251–9. Epub 2006/07/29. 10.1016/j.vetpar.2006.05.015 . 16872751

[111] Baig S, Damian RT, Molinari JL, Tato P, Morales-Montor J, Welch M, et al Purification and characterization of a metacestode cysteine proteinase from *Taenia solium* involved in the breakdown of human IgG. Parasitology. 2005;131(Pt 3):411–6. Epub 2005/09/24. . 16178363 10.1017/s0031182005007821

[112] Wang Q, Zhang S, Luo X, Hou J, Zhu X, Cai X. Cloning and characterization of a cathepsin L-like cysteine protease from *Taenia pisiformis*. Vet Parasitol. 2013;194(1):26–34. Epub 2013/02/16. 10.1016/j.vetpar.2012.12.055 . 23411373

[113] Tato P, Fernandez AM, Solano S, Borgonio V, Garrido E, Sepulveda J, et al A cysteine protease from *Taenia solium* metacestodes induce apoptosis in human CD4+ T-cells. Parasitol Res. 2004;92(3):197–204. Epub 2003/12/04. 10.1007/s00436-003-1008-1 . 14652742

[114] Molinari JL, Mejia H, White AC Jr., Garrido E, Borgonio VM, Baig S, et al *Taenia solium*: a cysteine protease secreted by metacestodes depletes human CD4 lymphocytes in vitro. Exp Parasitol. 2000;94(3):133–42. Epub 2000/06/01. 10.1006/expr.2000.4490 . 10831377

[115] White AC Jr., Baig S, Chappell CL. Characterization of a cysteine proteinase from *Taenia crassiceps* cysts. Mol Biochem Parasitol. 1997;85(2):243–53. Epub 1997/04/01. . 9106197 10.1016/s0166-6851(96)02839-3

[116] Ambrosio J, Landa A, Merchant MT, Laclette JP. Protein uptake by cysticerci of *Taenia crassiceps*. Arch Med Res. 1994;25(3):325–30. Epub 1994/01/01. . 7803983

[117] Hayunga EG, Sumner MP, Letonja T. Evidence for selective incorporation of host immunoglobulin by strobilocerci of *Taenia taeniaeformis*. J Parasitol. 1989;75(4):638–42. Epub 1989/08/01. . 2760776

[118] Sako Y, Yamasaki H, Nakaya K, Nakao M, Ito A. Cloning and characterization of cathepsin L-like peptidases of *Echinococcus multilocularis* metacestodes. Mol Biochem Parasitol. 2007;154(2):181–9. Epub 2007/06/08. 10.1016/j.molbiopara.2007.04.016 . 17553577

[119] Sako Y, Nakaya K, Ito A. *Echinococcus multilocularis*: identification and functional characterization of cathepsin B-like peptidases from metacestode. Exp Parasitol. 2011;127(3):693–701. Epub 2010/11/26. 10.1016/j.exppara.2010.11.005 . 21095185

[120] Geldhof P, Visser A, Clark D, Saunders G, Britton C, Gilleard J, et al RNA interference in parasitic helminths: current situation, potential pitfalls and future prospects. Parasitology. 2007;134(Pt 5):609–19. Epub 2007/01/05. 10.1017/S0031182006002071 . 17201997

[121] Stefanic S, Dvorak J, Horn M, Braschi S, Sojka D, Ruelas DS, et al RNA interference in *Schistosoma mansoni* schistosomula: selectivity, sensitivity and operation for larger-scale screening. PLoS Negl Trop Dis. 2010;4(10):e850 Epub 2010/10/27. 10.1371/journal.pntd.0000850 . 20976050 PMC2957409

[122] Geldhof P, Murray L, Couthier A, Gilleard JS, McLauchlan G, Knox DP, et al Testing the efficacy of RNA interference in *Haemonchus contortus*. Int J Parasitol. 2006;36(7):801–10. Epub 2006/02/14. 10.1016/j.ijpara.2005.12.004 . 16469321

[123] Dalzell JJ, Warnock ND, McVeigh P, Marks NJ, Mousley A, Atkinson L, et al Considering RNAi experimental design in parasitic helminths. Parasitology. 2012;139(5):589–604. Epub 2012/01/06. 10.1017/S0031182011001946 . 22216952

[124] Maule AG, McVeigh P, Dalzell JJ, Atkinson L, Mousley A, Marks NJ. An eye on RNAi in nematode parasites. Trends Parasitol. 2011;27(11):505–13. Epub 2011/09/03. 10.1016/j.pt.2011.07.004 . 21885343

[125] Britton C, Murray L. Using *Caenorhabditis elegans* for functional analysis of genes of parasitic nematodes. Int J Parasitol. 2006;36(6):651–9. Epub 2006/04/18. 10.1016/j.ijpara.2006.02.010 . 16616144

[126] Ward JD. Rendering the Intractable More Tractable: Tools from *Caenorhabditis elegans* Ripe for Import into Parasitic Nematodes. Genetics. 2015;201(4):1279–94. Epub 2015/12/09. 10.1534/genetics.115.182717 . 26644478 PMC4676526

[127] Murray L, Geldhof P, Clark D, Knox DP, Britton C. Expression and purification of an active cysteine protease of *Haemonchus contortus* using *Caenorhabditis elegans*. Int J Parasitol. 2007;37(10):1117–25. Epub 2007/04/25. 10.1016/j.ijpara.2007.02.012 . 17451718

[128] Parkinson J, Mitreva M, Whitton C, Thomson M, Daub J, Martin J, et al A transcriptomic analysis of the phylum Nematoda. Nat Genet. 2004;36(12):1259–67. Epub 2004/11/16. 10.1038/ng1472 . 15543149

[129] Blaxter ML. Nematoda: genes, genomes and the evolution of parasitism. Adv Parasitol. 2003;54:101–95. Epub 2004/01/09. . 14711085 10.1016/s0065-308x(03)54003-9

[130] Collins JJ, Newmark PA. It’s no fluke: the planarian as a model for understanding schistosomes. PLoS Pathog. 2013;9(7):e1003396 Epub 2013/07/23. 10.1371/journal.ppat.1003396 . 23874195 PMC3715406

[131] Rink JC, Vu HT, Sanchez Alvarado A. The maintenance and regeneration of the planarian excretory system are regulated by EGFR signaling. Development. 2011;138(17):3769–80. Epub 2011/08/11. 10.1242/dev.066852 . 21828097 PMC3152929

[132] Scimone ML, Srivastava M, Bell GW, Reddien PW. A regulatory program for excretory system regeneration in planarians. Development. 2011;138(20):4387–98. Epub 2011/09/23. 10.1242/dev.068098 . 21937596 PMC3177309

[133] Collins JJ, Wang B, Lambrus BG, Tharp ME, Iyer H, Newmark PA. Adult somatic stem cells in the human parasite *Schistosoma mansoni*. Nature. 2013;494(7438):476–9. Epub 2013/02/22. 10.1038/nature11924 . 23426263 PMC3586782

[134] Robb SM, Ross E, Sanchez Alvarado A. SmedGD: the *Schmidtea mediterranea* genome database. Nucleic Acids Res. 2008;36(Database issue):D599–606. Epub 2007/09/21. 10.1093/nar/gkm684 . 17881371 PMC2238899

[135] Newmark PA, Sanchez Alvarado A. Not your father’s planarian: a classic model enters the era of functional genomics. Nat Rev Genet. 2002;3(3):210–9. Epub 2002/04/25. 10.1038/nrg759 . 11972158

[136] Reddien PW, Bermange AL, Murfitt KJ, Jennings JR, Sanchez Alvarado A. Identification of genes needed for regeneration, stem cell function, and tissue homeostasis by systematic gene perturbation in planaria. Dev Cell. 2005;8(5):635–49. Epub 2005/05/04. 10.1016/j.devcel.2005.02.014 . 15866156 PMC2267917

[137] Walker G, Houthoofd K, Vanfleteren JR, Gems D. Dietary restriction in *C*. *elegans*: from rate-of-living effects to nutrient sensing pathways. Mech Ageing Dev. 2005;126(9):929–37. Epub 2005/05/18. 10.1016/j.mad.2005.03.014 . 15896824

[138] Geldhof P, Molloy C, Knox DP. Combinatorial RNAi on intestinal cathepsin B-like proteinases in *Caenorhabditis elegans* questions the perception of their role in nematode biology. Mol Biochem Parasitol. 2006;145(1):128–32. Epub 2005/11/18. 10.1016/j.molbiopara.2005.09.016 . 16289355

[139] Hashmi S, Britton C, Liu J, Guiliano DB, Oksov Y, Lustigman S. Cathepsin L is essential for embryogenesis and development of *Caenorhabditis elegans*. J Biol Chem. 2002;277(5):3477–86. Epub 2001/11/15. 10.1074/jbc.M106117200 . 11707440

[140] Hashmi S, Zhang J, Oksov Y, Ji Q, Lustigman S. The *Caenorhabditis elegans* CPI-2a cystatin-like inhibitor has an essential regulatory role during oogenesis and fertilization. J Biol Chem. 2006;281(38):28415–29. Epub 2006/07/22. 10.1074/jbc.M600254200 . 16857685

[141] Goupil LS, Ivry SL, Hsieh I, Suzuki BM, Craik CS, O’Donoghue AJ, et al Cysteine and Aspartyl Proteases Contribute to Protein Digestion in the Gut of Freshwater Planaria. PLoS Negl Trop Dis. 2016;10(8):e0004893 Epub 2016/08/09. 10.1371/journal.pntd.0004893 . 27501047 PMC4976874

[142] Horn M, Jilkova A, Vondrasek J, Maresova L, Caffrey CR, Mares M. Mapping the pro-peptide of the *Schistosoma mansoni* cathepsin B1 drug target: modulation of inhibition by heparin and design of mimetic inhibitors. ACS Chem Biol. 2011;6(6):609–17. Epub 2011/03/08. 10.1021/cb100411v . 21375333

[143] Fanfrlik J, Brahmkshatriya PS, Rezac J, Jilkova A, Horn M, Mares M, et al Quantum mechanics-based scoring rationalizes the irreversible inactivation of parasitic *Schistosoma mansoni* cysteine peptidase by vinyl sulfone inhibitors. J Phys Chem B. 2013;117(48):14973–82. Epub 2013/11/08. 10.1021/jp409604n . 24195769

[144] Caffrey CR. Chemotherapy of schistosomiasis: present and future. Curr Opin Chem Biol. 2007;11(4):433–9. Epub 2007/07/27. 10.1016/j.cbpa.2007.05.031 . 17652008

[145] WHO. Preventative chemotherapy in human helminthiasis Coordinated use of anthelminthic drugs in human interventions: a manual for health professionals and programme managers. Geneva:2006.

[146] Vermeire JJ, Lantz LD, Caffrey CR. Cure of hookworm infection with a cysteine protease inhibitor. PLoS Negl Trop Dis. 2012;6(7):e1680 Epub 2012/07/18. 10.1371/journal.pntd.0001680 . 22802972 PMC3389033

[147] Vermeire JJ, Suzuki BM, Caffrey CR. Odanacatib, a Cathepsin K Cysteine Protease Inhibitor, Kills Hookworm In Vivo. Pharmaceuticals (Basel). 2016;9(3):39 Epub 2016/07/08. 10.3390/ph9030039 . 27384569 PMC5039492

[148] Gauthier JY, Chauret N, Cromlish W, Desmarais S, Duong LT, Falgueyret JP, et al The discovery of odanacatib (MK-0822), a selective inhibitor of cathepsin K. Bioorg Med Chem Lett. 2008;18(3):923–8. Epub 2008/01/30. 10.1016/j.bmcl.2007.12.047 . 18226527

[149] Ndao M, Beaulieu C, Black WC, Isabel E, Vasquez-Camargo F, Nath-Chowdhury M, et al Reversible cysteine protease inhibitors show promise for a Chagas disease cure. Antimicrob Agents Chemother. 2014;58(2):1167–78. Epub 2013/12/11. 10.1128/AAC.01855-13 . 24323474 PMC3910870

[150] WHO. Research Priorities for Helminth Infections: technical report of the TDR disease reference group on helminth infections. 2012. 23420950

[151] Williamson AL, Brindley PJ, Knox DP, Hotez PJ, Loukas A. Digestive proteases of blood-feeding nematodes. Trends Parasitol. 2003;19(9):417–23. Epub 2003/09/06. . 12957519 10.1016/s1471-4922(03)00189-2

[152] Loukas A, Bethony JM, Williamson AL, Goud GN, Mendez S, Zhan B, et al Vaccination of dogs with a recombinant cysteine protease from the intestine of canine hookworms diminishes the fecundity and growth of worms. J Infect Dis. 2004;189(10):1952–61. Epub 2004/05/04. 10.1086/386346 . 15122534

[153] Young ND, Jex AR, Li B, Liu S, Yang L, Xiong Z, et al Whole-genome sequence of *Schistosoma haematobium*. Nat Genet. 2012;44(2):221–5. Epub 2012/01/17. 10.1038/ng.1065 . 22246508

[154] Dvořák J, Mashiyama ST, Braschi S, Sajid M, Knudsen GM, Hansell E, et al Differential use of protease families for invasion by schistosome cercariae. Biochimie. 2008;90(2):345–58. Epub 2007/10/16. 10.1016/j.biochi.2007.08.013 . 17936488

[155] Hola-Jamriska L, Tort JF, Dalton JP, Day SR, Fan J, Aaskov J, et al Cathepsin C from *Schistosoma japonicum*—cDNA encoding the preproenzyme and its phylogenetic relationships. Eur J Biochem. 1998;255(3):527–34. Epub 1998/09/17. . 9738890 10.1046/j.1432-1327.1998.2550527.x

[156] Brindley PJ, Kalinna BH, Dalton JP, Day SR, Wong JY, Smythe ML, et al Proteolytic degradation of host hemoglobin by schistosomes. Mol Biochem Parasitol. 1997;89(1):1–9. Epub 1997/09/23. . 9297696 10.1016/s0166-6851(97)00098-4

[157] Caffrey CR, Salter JP, Lucas KD, Khiem D, Hsieh I, Lim KC, et al SmCB2, a novel tegumental cathepsin B from adult *Schistosoma mansoni*. Mol Biochem Parasitol. 2002;121(1):49–61. Epub 2002/05/03. . 11985862 10.1016/s0166-6851(02)00022-1

[158] Chung YB, Kong Y, Yang HJ, Kang SY, Cho SY. Cysteine protease activities during maturation stages of *Paragonimus westermani*. J Parasitol. 1997;83(5):902–7. Epub 1997/10/29. . 9379296

[159] Park H, Hong KM, Sakanari JA, Choi JH, Park SK, Kim KY, et al *Paragonimus westermani*: cloning of a cathepsin F-like cysteine proteinase from the adult worm. Exp Parasitol. 2001;98(4):223–7. Epub 2001/09/19. 10.1006/expr.2001.4634 . 11560415

[160] Wilson LR, Good RT, Panaccio M, Wijffels GL, Sandeman RM, Spithill TW. *Fasciola hepatica*: characterization and cloning of the major cathepsin B protease secreted by newly excysted juvenile liver fluke. Exp Parasitol. 1998;88(2):85–94. Epub 1998/04/16. 10.1006/expr.1998.4234 . 9538862

